# Mechanoluminescence from Organic–Inorganic Metal Halide Perovskite Derivative

**DOI:** 10.1002/advs.202414588

**Published:** 2025-01-14

**Authors:** Hongyuan Zhao, Xinyu Yang, Yunfei Bai, Qichao Meng, Ziying Wen, Haibo Sun, Qilin Wei, Dan Huang, William W. Yu, Feng Liu

**Affiliations:** ^1^ Institute of Frontier Chemistry School of Chemistry and Chemical Engineering Shandong University Qingdao 266237 P. R. China; ^2^ School of Chemistry and Chemical Engineering Ministry of Education Key Laboratory of Special Functional Aggregated Materials Shandong Key Laboratory of Advanced Organosilicon Materials and Technologies Shandong University Jinan 250100 P. R. China; ^3^ Shandong Provincial Key Laboratory for Science of Material Creation and Energy Conversion Science Center for Material Creation and Energy Conversion Shandong University Qingdao 266237 P. R. China; ^4^ School of Physical Science and Technology Guangxi University Nanning 530004 P. R. China

**Keywords:** lanthanide‐based perovskite derivatives, mechanoluminescence, metal halide perovskite derivatives, organic–inorganic hybrid metal halides

## Abstract

Metal halide perovskites and their derivatives are gaining significant attention as photoluminescent materials due to their exceptional light‐emitting properties. However, most research has concentrated on electroluminescence and photoluminescence, there remains a substantial gap in the exploration of mechanoluminescence (ML) properties in perovskites, making this field largely uncharted. ML is an ancient and intriguing luminescent phenomenon that occurs when a material is subjected to mechanical forces. Here, the discovery of the first organic–inorganic hybrid terbium (Tb^3+^)‐based metal halide, a type of perovskite derivative, which exhibits notable ML properties is reported. Through material orthogonal design, the critical roles played by molecular geometry and metal‐site ions in achieving remarkable ML in organic–inorganic hybrid metal halides are identified.

## Graphical abstract

An organic–inorganic hybrid terbium (Tb^3+^)‐based metal halide, a derivative of perovskite, is found to exhibit remarkable mechanoluminescence (ML) properties. Similar to organic crystals, molecular geometry is essential for perovskites to exhibit ML. Additionally, the type of atoms, which affects polarity by effecting charge distribution, also plays a crucial role in determining the presence of ML properties.

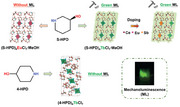

## 1 Introduction

When certain materials are subjected to stress, such as being rubbed, squeezed, or fractured, they can emit light without producing heat. This phenomenon is called mechanoluminescence (ML).^[^ ^1^, ^2^, ^3^ ^]^ ML continues to captivate researchers due to its promising applications in optics and its role in enhancing our fundamental understanding of material behavior under stress.^[^ ^4^, ^5^, ^6^ ^]^ Certain inorganic salts and organic molecules have been found to exhibit ML.^[^ ^7^ ^]^ However, inorganics often lacked sufficient brightness and frequently required high‐temperature calcination.^[^ ^8^, ^9^ ^]^ Organic crystals typically have higher luminescent intensity and the ability to be processed at lower temperatures.^[^ ^10^, ^11^ ^]^ However, achieving the desired crystalline structure and ML properties often necessitates specific processing techniques for organic crystals, which can be complex and challenging to scale up. Therefore, simplifying the research framework and developing user‐friendly materials are imperative.

Lead (Pb)‐based metal halide perovskites (MHPs) have been gaining considerable attention in recent years, which are prized for their elemental simplicity and the ease with which their crystal structure can be manipulated.^[^ ^12^, ^13^ ^]^ These materials exhibit excellent optical properties, including near 100% absolute photoluminescence quantum yield (PLQY), showing great potential for applications in LEDs, lasers, and photodetectors.^[^ ^14^ ^]^ However, despite their excellent PL properties, to the best of our knowledge, ML has not been reported in MHPs. Due to the diverse structures and compositions of MHPs, we aim to design an ML MHP, with the expectation of achieving special optical properties.

To design MHPs exhibiting ML, we can draw initial inspiration from asymmetric organic crystals. By screening all reported electromechanical luminescent materials, it appears that most compounds with ML belong to asymmetric crystals.^[^ ^2^ ^]^ Several recent studies suggest that the polarity of the organic crystals is a prerequisite for generating ML.^[^ ^10^, ^15^ ^]^ A polar crystal typically refers to a crystalline material that exhibits permanent electric polarization, which arises from the asymmetrical arrangement of its constituent atoms or molecules within the crystal structure. Such crystals often possess a non‐centrosymmetric structure, meaning that they lack a center of inversion symmetry. Asymmetric piezoelectric crystals, particularly polar ones, generate piezoelectricity under stress, which tilts the energy bands and decreases the depth of the trap levels, releasing carriers from the traps. The energy from carrier recombination is then transferred to the luminescent center, resulting in ML.^[^ ^16^ ^]^ Inspired by these, in this work, we focused on lanthanide‐based organic–inorganic MHPs, imparting them with polarity.

## 2 Results and Discussion

To obtain polar MHPs, chiral molecules are considered. Chiral molecules are mostly polar molecules, with their polarity arising due to asymmetrical structure. Therefore, we initiated our research with chiral compounds, specifically selecting terbium (Tb^3+^) MHPs as our study subjects. Tb^3+^ complexes typically exhibit fluorescence in the visible light range, which is advantageous for observing ML with the naked eye.

### 2.1 (R‐HPD)_4_TbCl_7_∙MeOH and (S‐HPD)_4_TbCl_7_∙MeOH

**Figure 1:**
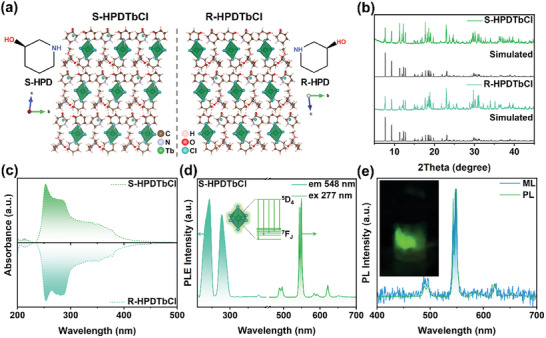
a) Crystal structure of R/S‐HPDTbCl and schematic diagrams of R/S‐HPD. b) Actual and simulated PXRD patterns for R/S‐HPDTbCl. c) UV–vis absorption, d) PL and PLE spectra of R/S‐HPDTbCl. e) PL and ML signal of S‐HPDTbCl. Inset: ML effect of S‐HPDTbCl upon scraping with a glass rod in dark.

Single crystals were synthesized using an antisolvent‐assisted recrystallization method (experimental details can be found in Supporting Information, SI). R/S‐(C_5_H_11_NO) (R/S‐(3‐hydroxy piperidine), abbreviated as R/S‐HPD), a chiral organic molecule (**Figure** **1**a), was employed as both a spacer cation and a chirality source in the synthesis. Such kind of molecule has also been used to produce Mn^2+^‐based MHPs with chirality.^[^ ^17^ ^]^ Crystal structures of the resulting (R/S‐HPD)_4_TbCl_7_∙MeOH (denoted as R/S‐HPDTbCl, MeOH: methanol) were determined by single‐crystal X‐ray diffraction (SCXRD), with the detailed crystal cell parameters provided in Tables S1, S2 (Supporting Information). R‐HPDTbCl and S‐HPDTbCl with mirror‐symmetric structures crystallize in the triclinic *P*1 space group. Powder XRD (PXRD) patterns of the R/S‐HPDTbCl enantiomers closely correspond to their simulated counterparts, confirming high phase purity (Figure 1b). The differences in the diffraction peak intensity ratios between the experimental and simulated PXRD patterns can be ascribed to the sample preparation process, particularly the grinding step. This process introduces variations in the orientation of the exposed crystal planes within the powdered sample, resulting in discrepancies in the XRD peak intensities.^[^ ^18^ ^]^ Elemental mapping images acquired by energy‐dispersive X‐ray spectroscopy (EDS) show a uniform distribution of Tb and Cl (Figure S1, Supporting Information), with an atomic ratio of 1:7 (Figure S2, Supporting Information), consistent with the SCXRD findings. X‐ray photoelectron spectroscopy (XPS) measurements further verified that Tb exists in the Tb^3+^ oxidation state (Figure S3, Supporting Information).

UV–vis absorption spectra of the two crystals (Figure 1c) showed two distinct absorption bands at 200–250 and 250–400 nm, attributable to the charge transfer band of Cl^−^→Tb^3+^ and the parity‐allowed Tb^3+^ 4*f*‐5*d* transitions, respectively. Both emitted green PL at room temperature, with high PLQYs of ≈100% and PL lifetime of ≈5 ms (Figure S4, Supporting Information). We note that the PLQY achieved by our material is significantly higher than that of previously reported ML materials (see Table S3, Supporting Information).PL spectra depicted in Figure 1d closely resemble the characteristic luminescence of Tb^3+^, indicating that the Tb atom serves as the luminescent center.

So far, PL properties of R/S‐HPDTbCl align with those of our previously documented Tb^3+^‐based MHPs, such as (DFPD)_4_TbCl_7_ and (DMA)_4_TbCl_7_.^[^ ^19^, ^20^ ^]^ However, unlike these materials, which lack ML, R/S‐HPDTbCl exhibits significant ML performance. When the two crystals were crushed with a glass sheet at room temperature, both exhibited bright green ML (Video S1, Supporting Information). Similarly, when the crystals were smashed using rods made from various materials such as metal, crabstick, or Teflon, ML emissions were still observed (Video S2, Supporting Information), indicating that this process is independent of the type of stimulus source, similar to those of organic molecules.^[^ ^8^ ^]^ ML spectrum of R/S‐HPDTbCl shows an emission signal identical to that of PL (Figure 1e), suggesting that the luminous center of ML remains associated with the Tb^3+^ ions.

To investigate the triggering conditions of ML, we placed the crystals on a digital pressure testing machine and recorded the entire process, from the application of force to the fracture of the crystals (Video S3, Supporting Information), observing that ML was generated only at the moment of crystals fracture, with no ML occurring afterward, despite the continued application of force. Although ML can be generated during crystal fracture, this effect is irreversible. In this regard, we dissolved the deactivated powder in a small amount of MeOH and reheated it, revealed that the recrystallized powder still had ML properties (Video S4, Supporting Information), thus enhancing the reversibility for potential practical applications. Considering that the ML properties of the crystalline powders are still retained, we combined the milled powder with styrene‐ethylene‐butylenestyrene (SEBS) to form polymer‐based composite film (Figure S5, Supporting Information), which still exhibited ML properties under pressure (Video S5, Supporting Information), promising to realize the potential applications of stress detection, structural damage monitoring, and anti‐counterfeiting encryption.

**Figure 2:**
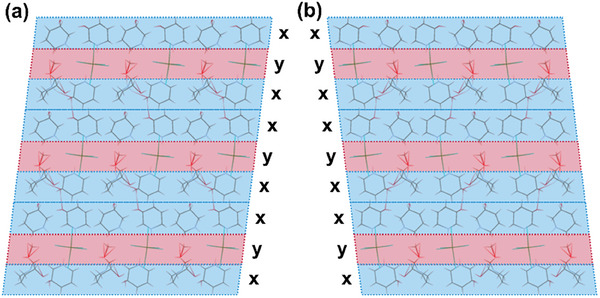
Packing modes of crystal a) R‐HPDTbCl and b) S‐HPDTbCl.

To better understand the ML properties of R/S‐HPDTbCl, detailed investigations into the molecular geometry were undertaken. A crucial lesson from organic crystals indicates that molecular packing plays a pivotal role in ML.^[^ ^15^ ^]^ To simplify the packing analysis and provide a clear description, three adjacent molecules were selected as a repeating unit. As shown in **Figure** **2**a,b, with molecules in crystal divided into two parts (*x* and *y*) characterized by markedly different packing modes, the distinct chemical environments of these zones were clearly observable. Molecules in the *x* region were surrounded by molecules from both the *x* and *y* parts. This resulted in crystal R/S‐HPDTbCl exhibiting an inhomogeneous system with varying strengths of intermolecular interactions. When a mechanical force was applied to the crystal, it easily fractured at weak points to form crack surfaces. These fractures could generate electrical potentials through the movement of charged dislocations. During the fracture process, new charged surfaces are created, resulting in a strong electric field. In the case of R/S‐HPDTbCl crystals, the piezoelectricity of R/S‐HPDTbCl makes it easy to generate an electric field. Due to the lack of a symmetric center for piezoelectric effects in the molecular packing, charge accumulation on crack surfaces intensified electron bombardments and even directly excites the luminescent center, thereby enhancing the likelihood of exhibiting ML properties.

### 2.2 (4‐HPD)_4_TbCl_7_

**Figure 3:**
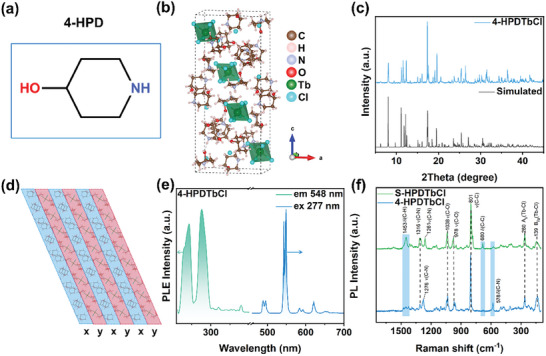
a) Schematic diagram of 4‐HPD. b) Crystal structure of 4‐HPDTbCl. c) Actual and simulated PXRD patterns for 4‐HPDTbCl. d) Packing mode, e) PL and PLE spectra of 4‐HPDTbCl. f) Raman spectra of S‐HPDTbCl and 4‐HPDTbCl.

To test the theory that molecular polarity is essential for generating ML in MHPs, we used a non‐chirality isomer of R/S‐HPD to construct non‐polar MHPs. 4‐hydroxy piperidine (4‐HPD), a non‐chirality molecule (**Figure** **3**a), was selected. SCXRD analysis revealed that the 4‐HPD^+^ cation also allowed the formation of 0D connected [TbCl_6_], ^3−^ although the resulting crystal (4‐HPD)_4_TbCl_7_ (referred to as 4‐HPDTbCl) did not establish coordination bonds with MeOH and crystallized in a different monoclinic phase with the *P*2_1_ */n* space group (Figure 3b and Table S4, Supporting Information). XRD measurement also confirmed their high phase purity and uniformity (Figure 3c). A similar investigation of the molecular geometry of 4‐HPDTbCl revealed a homogeneous chemical environment for each molecule (Figure 3d), with molecules in the *y*(*x*) region primarily surrounded by those from the *x*(*y*) region, confirming its classification as a non‐polar crystal. The resulting crystal exhibited PL/PLE signals similar to those of R/S‐HPDTbCl (Figure 3e). However, no ML was observed when they were scraped or ground (Video S6, Supporting Information), consistent with our previous assumptions.

To gain a deeper understanding of the distinct ML effect observed in R/S‐HPDTbCl and 4‐HPDTbCl, Raman measurements were further conducted. Variations in Raman shift or changes in peak intensity typically indicate differences in molecular concentration, chemical environments, or structural characteristics among the compounds. The analysis of the Raman spectra revealed that R/S‐HPDTbCl and 4‐HPDTbCl exhibited similar peaks assignable to the stretching vibrations of Tb‐Cl, C‐H, C‐N, and C‐C bands (Figure 3f). However, there were significant differences in peak intensity at several positions, such as 578 and 1453 cm^−1^. Additionally, the noticeable signal at 680 cm^−1^ for R/S‐HPDTbCl was largely absent in 4‐HPDTbCl, suggesting unique local environments in the two compounds. Such observation aligns with the analysis of their distinct molecular geometries, as illustrated in Figures 2 and 3d.

### 2.3 (S‐HPD)_4_EuCl_7_∙MeOH

**Figure 4:**
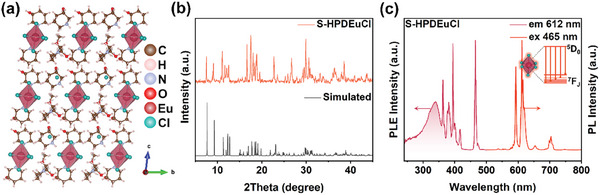
a) Crystal structure of S‐HPDEuCl. b) Actual and simulated PXRD patterns for S‐HPDEuCl. c) PL and PLE spectra of S‐HPDEuCl.

Our scientific inquiry continues. To investigate whether molecular geometry is the sole determinant of ML generation, we further refined the composition of metal elements in MHPs. Europium (Eu^3+^) was first used to replace Tb^3+^ because it can also emit visible light during radiative transitions. (S‐HPD)_4_EuCl_7_∙MeOH (denoted as S‐HPDEuCl) single crystals were prepared using the same method designed for S‐HPDTbCl. XRD analysis revealed that the crystal had exactly the same crystal structure as S‐HPDTbCl, and it also exhibited high purity and uniformity (**Figure** **4**a,b and Table S5, Supporting Information). Given the ML behavior of S‐HPDTbCl, we would have expected to observe similar phenomena in S‐HPDEuCl. However, despite identical molecular geometry, these crystals did not exhibit any observable ML. Instead, they only displayed PL originating from Eu^3+^ (Figure 4c and Video S7, Supporting Information), similar to that of non‐polar 4‐HPDTbCl.

To explain this phenomenon, we must consider whether molecular geometry is the sole factor determining a molecule's polarity. The answer is clearly no, as the electronegativity of the constituent elements also plays a significant role in influencing polarity through its effect on charge distribution. We calculated the Bader charges for both crystal structures to analyze the charge transfer states in the EuCl_6_ and TbCl_6_ octahedral structures. Theoretical calculation results (Supplementary Discussion 1) suggest that Eu has a higher electronegativity than Tb and the electrons gained by Cl atoms surrounding EuCl_6_ exhibit a lower degree of asymmetry compared to those surrounding TbCl_6_, suggesting that the polarity of the EuCl_6_ octahedral structure is lower than that of the TbCl_6_ structure. Piezoresponse force microscopy (PFM) was employed to evaluate the piezoelectric properties of S‐HPDTbCl, S‐HPDEuCl, and 4‐HPDTbCl crystals. The results revealed distinct piezoelectric hysteresis and butterfly loops for S‐HPDTbCl and S‐HPDEuCl, indicating their piezoelectric characteristics, whereas 4‐HPDTbCl exhibited no piezoelectric properties (Figure S8, Supporting Information). Notably, the phase shift and butterfly loop amplitude of S‐HPDTbCl were significantly stronger than those of S‐HPDEuCl, indicating that S‐HPDTbCl exhibits enhanced piezoelectric performance, which aligns well with our theoretical calculation results.

### 2.4 Eu^3+^‐, Ce^3+^‐, and Sb^3+^‐doped S‐HPDTbCl

Since metal elements can influence the luminescence of ML, we were next curious whether doping might alter ML properties of Tb^3+^ MHPs. In addition to Eu^3+^, two other luminescent dopants, cerium (Ce^3+^) and antimony (Sb^3+^), were employed. Theoretical calculations suggest that these dopants prefer to substitute the Tb site rather than undergo interstitial doping (Supplementary Discussion 2). SEM‐EDS, PXRD, and the inductively coupled plasma‐mass spectrometry confirmed the successful doping of these ions in S‐HPDTbCl (Figures S9, S10 and Table S8, Supporting information). Similar to our previously reported (DMA)_4_TbCl_7_, these doped crystals exhibited excitation wavelength‐dependent PL property (Figure S11, Supporting information). The PLE spectra monitored at the PL position of Tb^3+^ and dopant ions displayed distinct features, confirming the presence of two independent luminescent centers (Figure S12, Supporting information). Significantly, taking Eu^3+^‐doped crystals as an example, while the Eu atom occupies the Tb site and displays compound PL, these crystals did not emit the same light when they were scraped or ground; instead, they still emitted green light attributable to Tb^3+^ (Video S8, Supporting Information). Similar observations can be noted in scenarios involving Ce^3+^ and Sb^3+^ doping, where the ML phenomenon is exclusively associated with Tb^3+^ (Videos S9, S10, Supporting Information). These findings unambiguously confirm that the ML properties of MHPs are also closely dependent on the metal‐site ions.

## 3 Conclusions

ML properties were experimentally investigated in lanthanide‐based organic–inorganic MHPs. Through an orthogonal design of material structure and composition, we found that similar to organic crystals, molecular geometry is essential for perovskites to exhibit ML. However, unlike organic crystals, the type of atoms occupying the metal sites in perovskites, which affects polarity through its effect on charge distribution, also plays a crucial role in determining the presence of ML properties.

## Conflict of Interest

The authors declare no conflict of interest.

## Supporting information



Supporting Information

Supplemental Movie 1

Supplemental Movie 2

Supplemental Movie 3

Supplemental Movie 4

Supplemental Movie 5

Supplemental Movie 6

Supplemental Movie 7

Supplemental Movie 8

Supplemental Movie 9 Supplemental Movie 10

## Data Availability

The data that support the findings of this study are available from the corresponding author upon reasonable request.
