# Supplementary material for: Mechanoluminescence from Organic–Inorganic Metal Halide Perovskite Derivative
Source: Adv Sci (Weinh). 2025 Jan 14;12(9):2414588. doi: 10.1002/advs.202414588 (PMC11884580; doi:10.1002/advs.202414588)
Supplement: Supplementary file 1 — Supporting Information [file ADVS-12-2414588-s005.docx]

**Supporting Information**

**Mechanoluminescence from Organic-Inorganic Metal Halide Perovskite Derivative**

Hongyuan Zhao,^1^ Xinyu Yang,^1^ Yunfei Bai,^1^ Qichao Meng,^1^ Ziying Wen,^1^ Haibo Sun,^1^ Qilin Wei,^2^ Dan Huang,^3^ William W. Yu,*^,2^ Feng Liu*^,1^

*Hongyuan Zhao, Xinyu Yang, Yunfei Bai, Qichao Meng, Ziying Wen, Haibo Sun, Qilin Wei, Dan Huang, William W. Yu,* and Feng Liu**

H. Zhao, X. Yang, Y. Bai, Q. Meng, Z. Wen, H. Sun, F. Liu

Institute of Frontier Chemistry

School of Chemistry and Chemical Engineering

Shandong University

Qingdao 266237, P. R. China

E-mail: [fenglau189@sdu.edu.cn](mailto:fenglau189@sdu.edu.cn)

Q. Wei, W. W. Yu

School of Chemistry and Chemical Engineering

Ministry of Education Key Laboratory of Special Functional Aggregated Materials

Shandong Key Laboratory of Advanced Organosilicon Materials and Technologies

Shandong University

Jinan 250100, P. R. China

E-mail: [wyu6000@gmail.com](mailto:wyu6000@gmail.com)

Q. Wei, W. W. Yu

Shandong Provincial Key Laboratory for Science of Material Creation and Energy Conversion

Science Center for Material Creation and Energy Conversion

Shandong University

Qingdao 266237, P. R. China

D. Huang

School of Physical Science and Technology

Guangxi University

Nanning 530004, P. R. China

**1. Experimental Details**

**1.1 Materials**

(R/S)-3-hydroxypiperidine hydrochloride (R/S-HPD·HCl, 98%, Aladdin), 4-hydroxypiperidine hydrochloride (4-HPD·HCl, > 98%, Aladdin), terbium chloride hexahydrate (TbCl_3_·6H_2_O, 99.9%, Macklin), europium chloride hexahydrate (EuCl_3_·6H_2_O, 99.99%, Aladdin), cerous chloride (CeCl_3_, 99.99%, Aladdin), antimony acetate (Sb(OAc)_3_, 99.99%, Macklin), hydrochloric acid (HCl, 37% in water, Sinopharm Chemical Reagent Co., Ltd, China), diethyl ether (≥ 99.7%, Sinopharm Chemical Reagent Co., Ltd, China), methanol (MeOH, 99.5%, Aladdin), toluene (99.5%, Sinopharm Chemical Reagent Co., Ltd, China), and styrene-ethylene-butylenestyrene (SEBS G1660, Kraton) were used as received without further purification.

**1.2 Synthesis of** **(R/****S-HPD)_4_TbCl_7_∙MeOH,** **(S-HPD)_4_EuCl_7_∙MeOH, and (4-HPD)_4_TbCl_7_ single crystals**

All the single crystals were synthesized with a similar procedure. In a typical procedure, a 25 mL round bottom flask was charged with 2 mmol R/S-HPD·HCl and 0.5 mmol TbCl_3_·6H_2_O. 1.5 mL of HCl was added and the mixture was heated to 130 °C for about 3 hours until a dried solid powder formed. The solid powder was redissolved in 1 mL of MeOH, which was then transferred to an open cuvette and placed in a sealed vial containing 10 mL of diethyl ether. The vial was left undisturbed for 4 days to obtain transparent R/S-HPD_4_TbCl_7_·MeOH single crystals. Synthesis of S-HPD_4_EuCl_7_·MeOH and 4-HPD_4_TbCl_7_ single crystals were performed in a similar way, where 0.5 mmol EuCl_3_·6H_2_O and 2 mmol 4-HPD·HCl were used, respectively.

**1.3 Synthesis of Ce^3+^/Eu^3+^/Sb^3+^-doped (S-HPD)_4_TbCl_7_·MeOH single crystals**

In a typical procedure, a 25 mL round bottom flask was charged with 2 mmol R/S-HPD·HCl, 0.5 mmol TbCl_3_·6H_2_O and 0.05 mmol CeCl_3_ (EuCl_3_·6H_2_O or Sb(OAc)_3_). 1.5 mL of HCl was added and the mixture was heated to 130 °C for ~3 hours until a dried solid powder formed. The solid powder was redissolved in 1 mL of MeOH, which was then transferred to an open cuvette and placed in a sealed vial containing 10 mL of diethyl ether. The vial was left undisturbed for 4 days to grow transparent Ce^3+^/Eu^3+^/Sb^3+^-doped (S-HPD)_4_TbCl_7_·MeOH single crystals.

**1.4** **Synthesis of SEBS-based composite film**

First, SEBS was dissolved in toluene with a mass fraction of 20 % under magnetic stirring at room temperature until total dissolution of the polymer. Then, 35% by mass of R/S-HPDTbCl power according to the mass of SEBS was slowly added to the solution under magnetic stirring. Finally, the prepared solution was injected into the mold and left to stand for one day, after which the SEBS-based composite film was removed from the mold.

**2. Characterizations**

Powder X-ray diffraction (XRD) measurements were performed on a Rigaku SmartLab 9 kW diffractometer equipped with Cu Kα radiation (Rigaku Corp., Japan). UV-vis absorption spectra were acquired with a Shimadzu UV-2600i spectrophotometer. The inductively coupled plasma-mass spectrometry (ICP-MS) measurements were conducted using Perkin-Elmer ICP-MS NexION 300X. X-ray photoelectron spectroscopy (XPS) data were accumulated on a photoelectron spectrometer (Thermo Fisher ESCALAB Xi+) operated at 200 W. A monochromatic Al *K*_α_ X-ray source (1486.6 eV) is used as the excitation source in all cases. All spectra were recorded using an entrance aperture of 300 × 700 μm with a pass energy of 80 eV for wide scans and 20 eV for high-resolution scans. The instrument sensitivity was 7.5 × 10^5^ counts/s when measuring the Ag 3d5/2 photoemission peak for a clean Ag sample recorded at a pass energy of 20 eV and 450 W emission power. The absolute error in the acquisition of binding energies is ~0.1 eV, as quoted by the instruments manufacturer. XPS data were analyzed using Thermo Scientific Advantage Data System software, and all spectra were referenced to the C 1s peak (284.5 eV). Peaks were fitted using GL(30) line shapes, a combination of Gaussian (70%) and Lorentzian (30%). The full width at half-maximum (FWHM) of each component was initially constrained to 0.8~1.5 eV. UV-vis absorption spectra were recorded with a spectrophotometer (HITACHI, U-3900H, Japan). The absolute photoluminescence (PL) quantum yield was measured by using an integrating sphere on an Absolute PL Quantum Yield Spectrometer C11347 at an excitation power of 0.1 mW (Hamamatsu, Japan). Time-resolved PL spectra were measured using a FLS1000 Edinburgh Instruments spectrofluorometer equipped with the integrating sphere. Energy-dispersive X-ray spectroscopy (EDS) elemental mapping images were obtained using FEI Quanta 250 FEG environmental scanning electron microscope equipped with a Bruker energy dispersive X-ray spectrometer QUANTAX 200 with XFlash®6 detector. Mechanoluminescence (ML) spectrum was measured using a PMA-12 photonic multichannel analyzer (C10027-01, Hamamatsu, Japan). The Raman data were collected using the Micro-Raman Spectrometer inVia Qontor (Renishaw) under the excited wavelength of 785 nm with an exposure time of 30 s and the number of accumulations 5. Piezo-response force microscopies (PFM) were recorded by an atomic force microscope (Bruker Dimension Icon with a SCM-PIT, Pt/Ir-coated tips) with contact mode.

**3. Calculation Details**

The first-principles calculations were carried out based on the density functional theory (DFT) as implemented in the Vienna ab initio simulation package (VASP)^[1, 2]^. The projector augmented wave (PAW) method^[3]^ was used to represent the interactions of the core-valence electrons. Exchange and correlation potentials were treated in the generalized gradient approximation (GGA) of the Perdew Burke Ernzerhof (PBE)^[4]^. A kinetic energy cutoff of 500 eV was used for the plane wave expansion. MonkhorstPack^[5]^ k-point sampling method with the K-spacing value of 0.03 2π/Å was used for the Brillouin zone integration. DFT-D3 with the BJ-damping method developed by Grimme^[6]^ was included to consider the van der Waals interactions. During structure relaxation, the convergence criteria for the total energy and force were 1.0 ×10^-6^ eV and 0.05 eV/Å.

**Calculation of the formation energy (*E*_f_):** The *E*_f_ for substitution doping condition was calculated by formula:

*E*_f_ = *E*_tot_(HPDTbCl + dopant) – *E*_tot_(HPDTbCl) + *μ*_Tb_ – *μ*_dopant_ (1)

For interstitial doping, *E*_f_ value was calculated by formula:

*E*_f_ = *E*_tot_(HPDTbCl + Ce) – *E*_tot_(HPDTbCl) – *μ*_dopant_ (2)

where *E*_tot_(HPDTbCl + dopant) and *E*_tot_(HPDTbCl) are the total energy of the doped HPDTbCl system and the pristine HPDTbCl crystal, respectively. *μ*_Tb_ and *μ*_dopant_ are the chemical potential of Tb and dopant atom, respectively, calculated as the corresponding crystal energy per atom.

**Calculation of the Bader charges:** Ultra-soft pseudopotentials were applied for all elements, including H, C, N, O, Cl, Eu, and Tb. A kinetic energy cutoff of 400 eV was set, along with a 4×4×4 Monkhorst–Pack k-point grid for the wavefunction basis. Structural relaxations followed an energy convergence threshold of 1.0×10^-^⁴ eV. The Bader charges in the structure were calculated by subtracting the valence charge from each atom’s total charge.


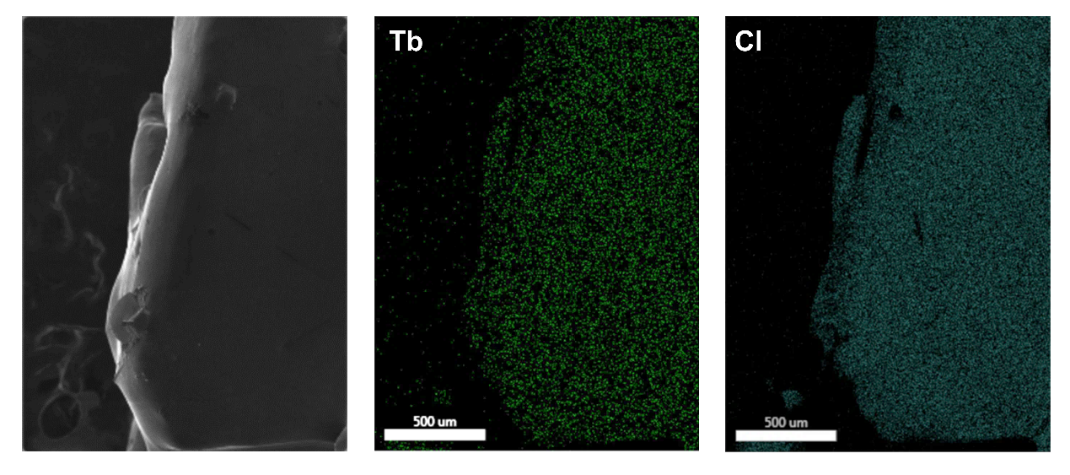


**Figure S1.** SEM/EDS elemental maps of S-HPDTbCl crystals.


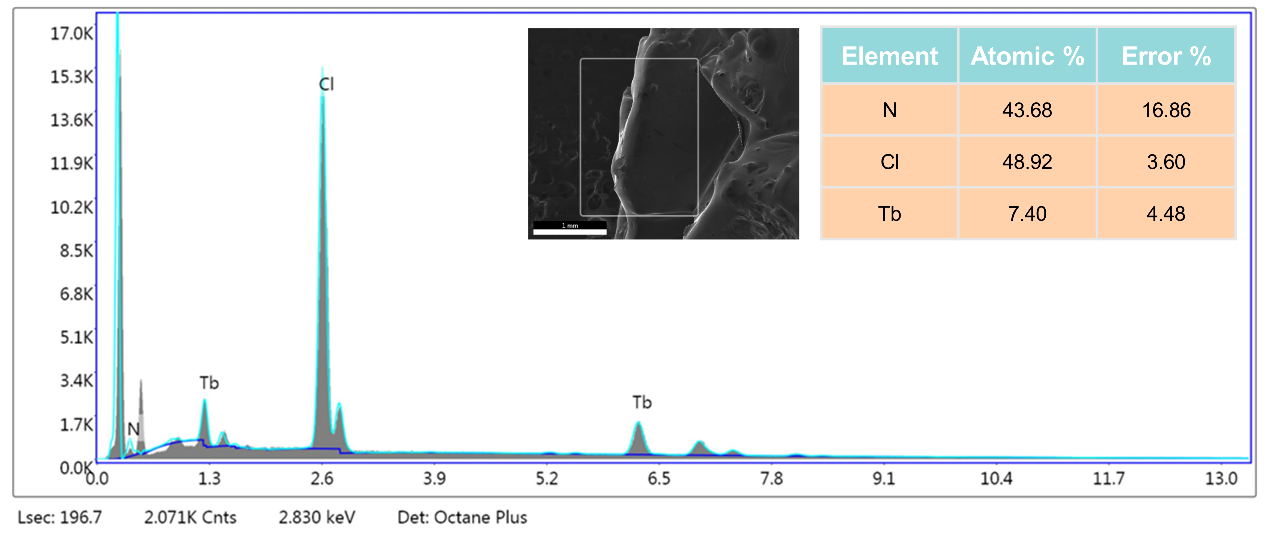


**Figure S2.** SEM/EDS quantitative analysis of S-HPDTbCl crystals.


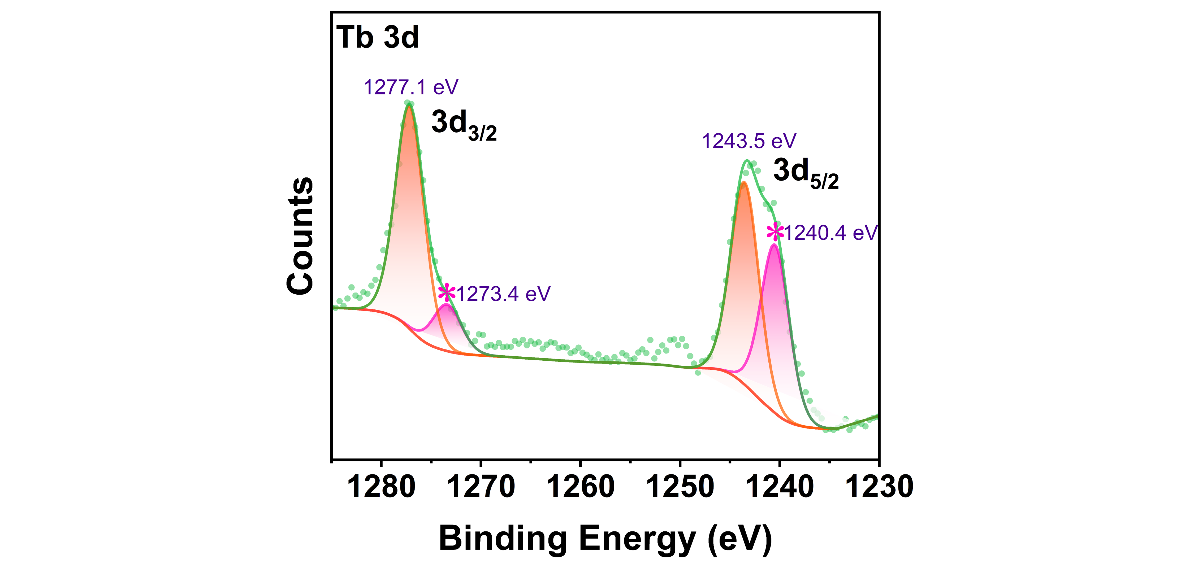


**Figure S3.** XPS spectra of Tb 3d of S-HPDTbCl crystals. The doublet structure of Tb (for 3d_5/2_ and 3d_3/2_, respectively) is observed at binding energies of 1243.5 and 1277.1 eV, accompanied by typical shake-down satellite peaks at 1240.4 and 1273.4 eV (marked with asterisks).


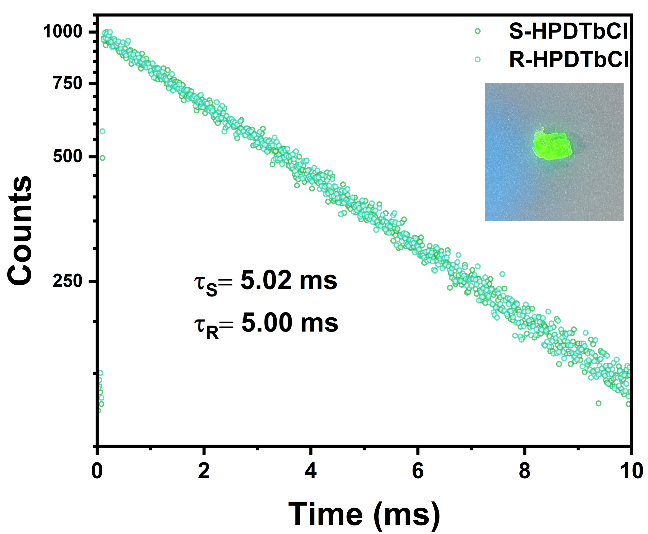


**Figure S4.** PL decay curves of R/S-HPDTbCl crystals. Inset shows a picture of a typical S-HPDTbCl crystal excited under 365 nm UV light.


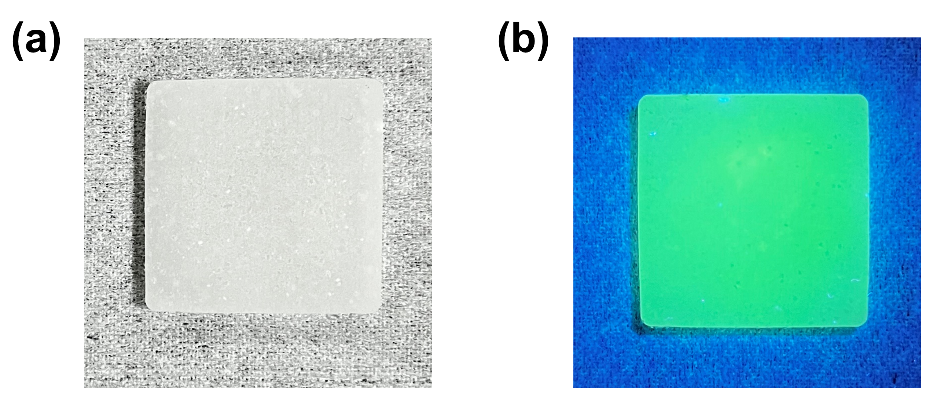


**Figure S5.** Images of SEBS-based composite film (a) in daylight and (b) under 365 nm.

**Supplementary Discussion 1**


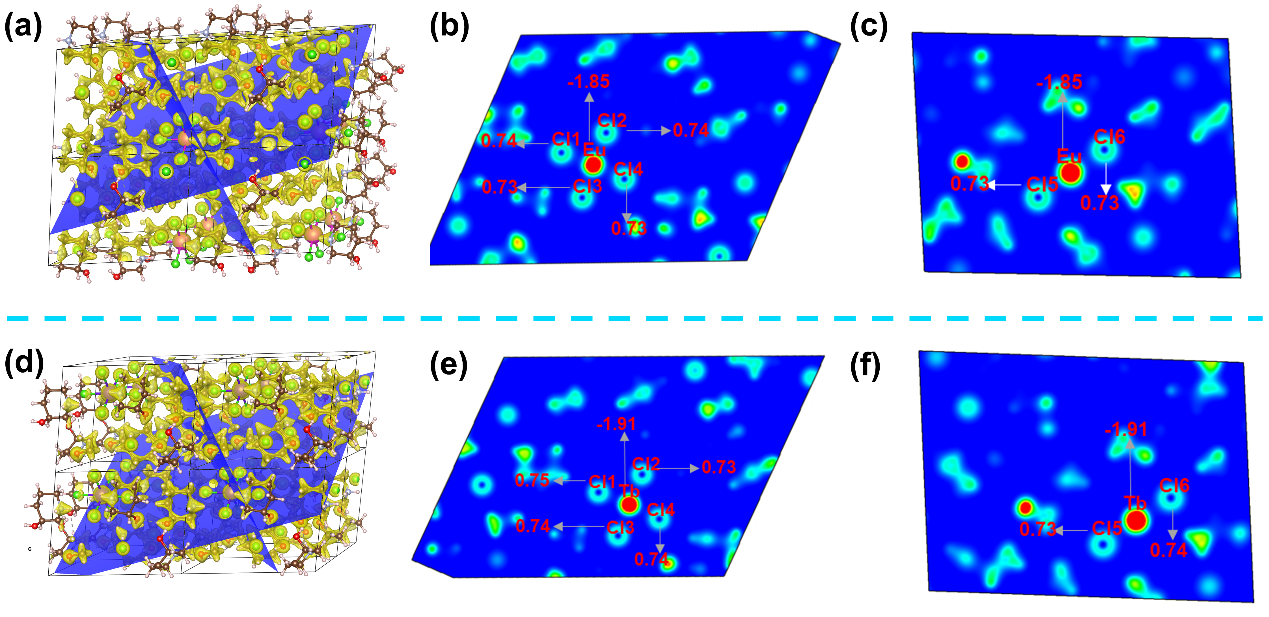


**Figure S6.** (a) Charge density distribution of the S-HPDEuCl structure. (b-c) Two-dimensional cross-sectional charge distribution of EuCl_6_ with the labeled electron donation and acceptance. (d) Charge density distribution of the S-HPDTbCl structure. (e-f) Two-dimensional cross-sectional charge distribution of TbCl_6_ with the labeled electron donation and acceptance.

We calculated the Bader charges for both crystal structures to analyze the charge transfer states in the EuCl_6_ and TbCl_6_ octahedral structures. The specific data can be found in Table S5-6. Figure S5b-c correspond to two cross-sections of Figure S5a, while Figure S5e-f correspond to two cross-sections of Figure S5d. As shown in Figure S5b-c, in the octahedral structure of EuCl_6_, the Eu atom donates electrons, and the surrounding six Cl atoms are in a state of accepting electrons. Notably, the number of electrons accepted by the Cl atoms comes not only from the Eu atom but also from the electrons provided by the organic molecules. In comparison, in the EuCl_6_ octahedral structure, the number of electrons donated by the Eu atom is less than that donated by the Tb atom, indicating that the electronegativity of Eu is higher than that of Tb. Further analysis of the electron gained by Cl atoms surrounding EuCl_6_ reveals a lower degree of asymmetry than the Cl atoms surrounding TbCl_6_, suggesting that the polarity of the EuCl_6_ octahedral structure is lower than that of the TbCl_6_ structure.

**Supplementary Discussion 2**


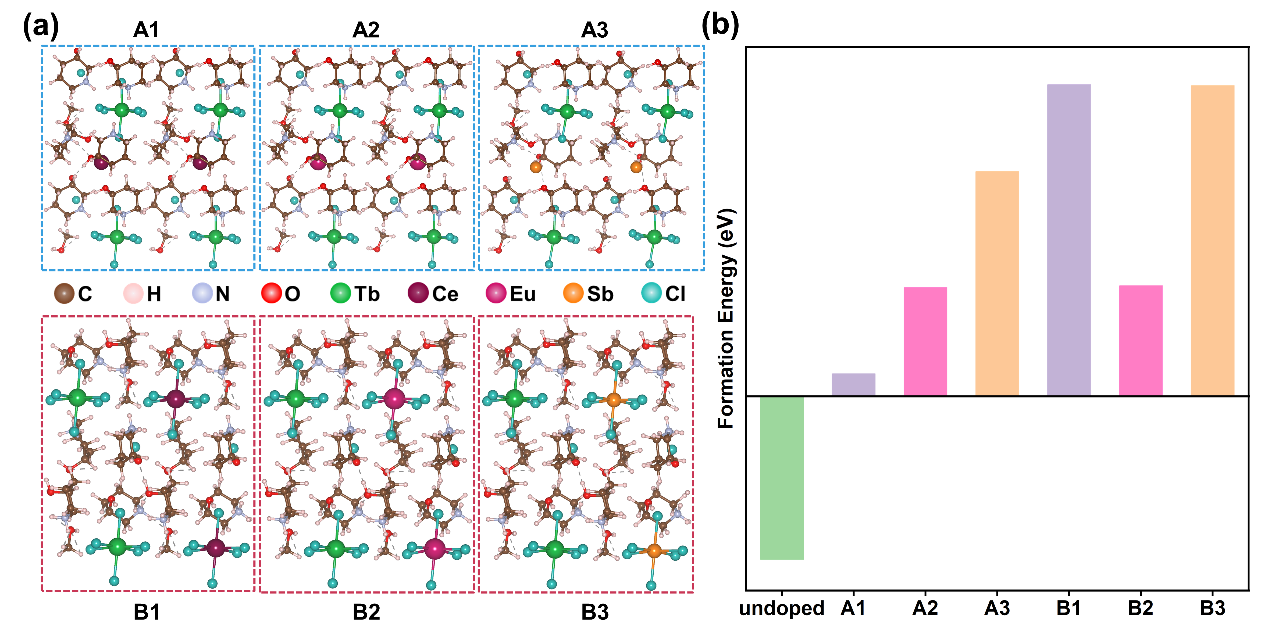


**Figure S7.** (a) Relaxed structures of possible doping conditions of S-HPDTbCl with Eu^3+^, Ce^3+^, and Sb^3+^ dopants. (b) Formation energies for different doping models. Model A: the substitution doping at Tb site; Model B: interstitial doping.

Based on DFT calculations, we investigated the possible atomic positions of Eu^3+^, Ce^3+^, and Sb^3+^ dopants in Tb crystals. Two potential doping scenarios, substitution (condition A) and interstitial (condition B), were examined. Figure S5a illustrates their relaxed structures and Figure S5b summarizes the corresponding formation energy (*E*_f_) values, including the undoped scenario. It is observed that substitution doping consistently yields lower *E*_f_ values than that of interstitial doping, suggesting that substitution at the Tb site is likely more feasible for synthesis compared to interstitial doping.

**
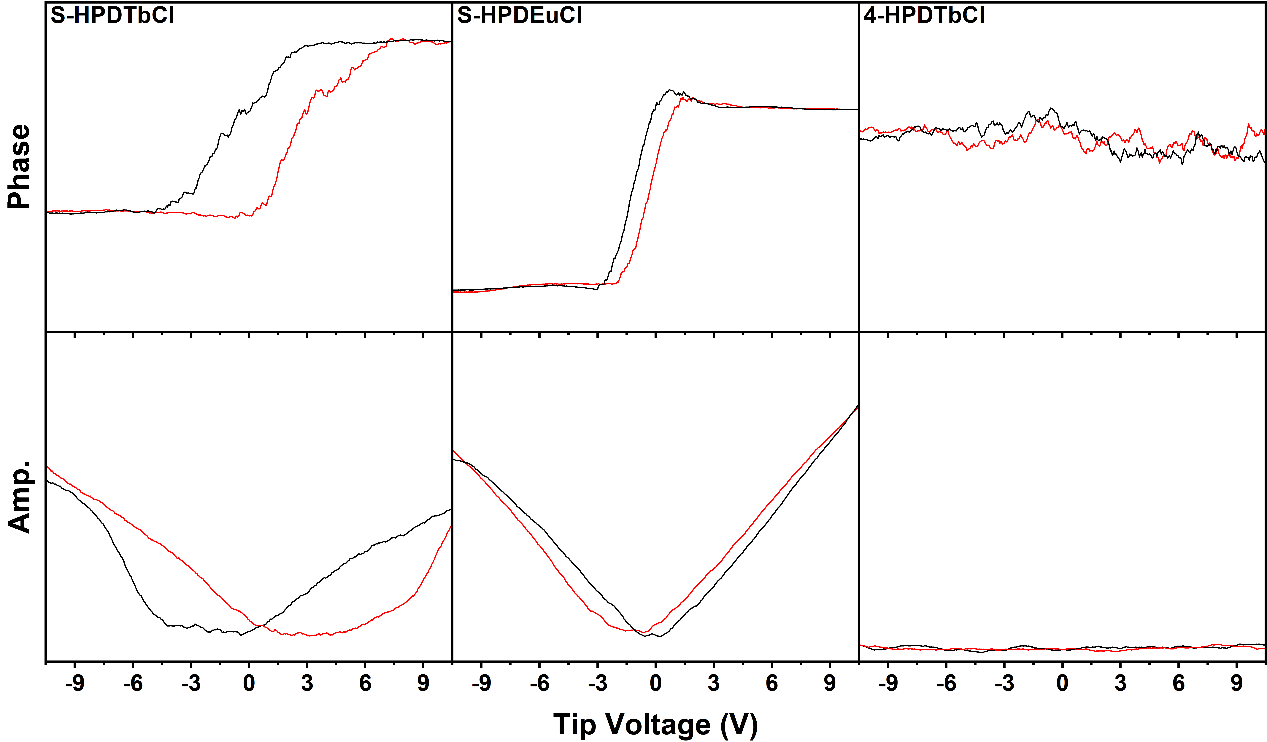
**

**Figure S8.** PFM curves of S-HPDTbCl, S-HPDEuCl, and 4-HPDTbCl crystals.


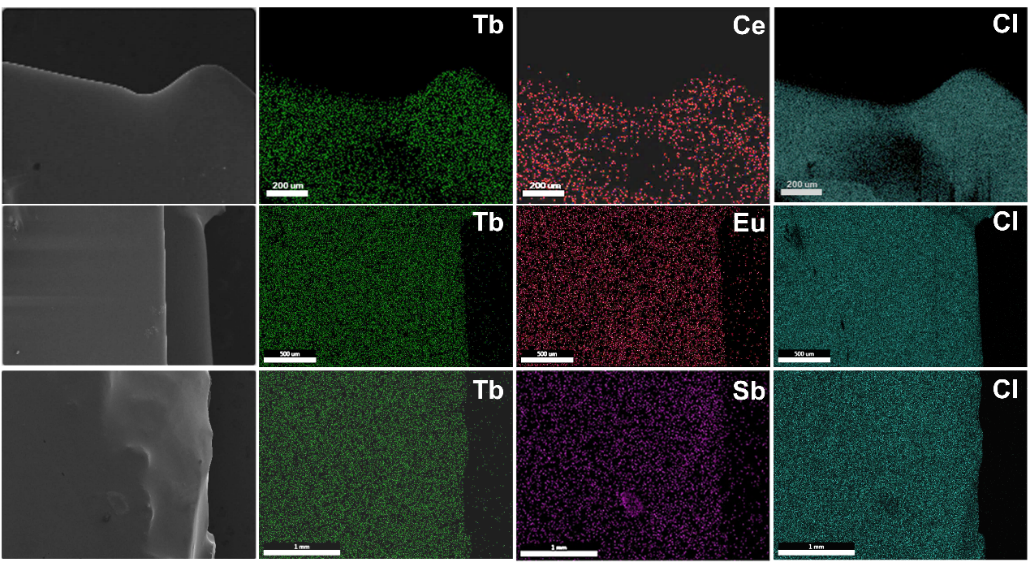


**Figure S9.** SEM/EDS elemental maps of S-HPDTbCl crystals doped with Ce^3+^, Eu^3+^, and Sb^3+^.


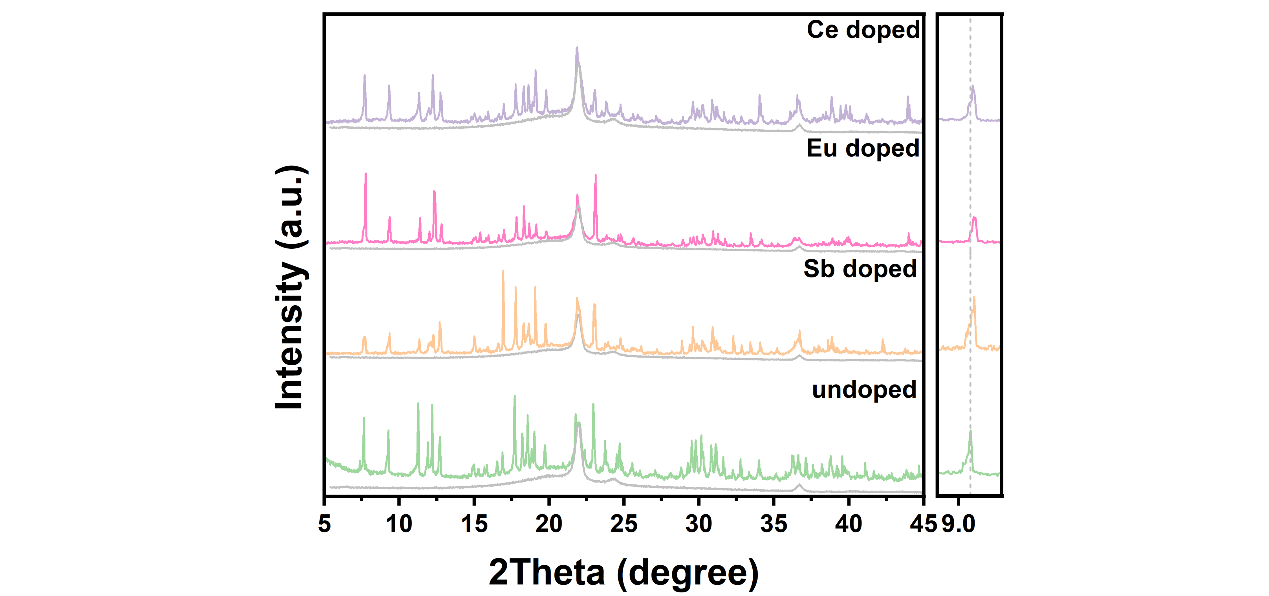


**Figure S10.** Powder XRD patterns of the S-HPDTbCl crystals doped with Ce^3+^, Eu^3+^, and Sb^3+^.


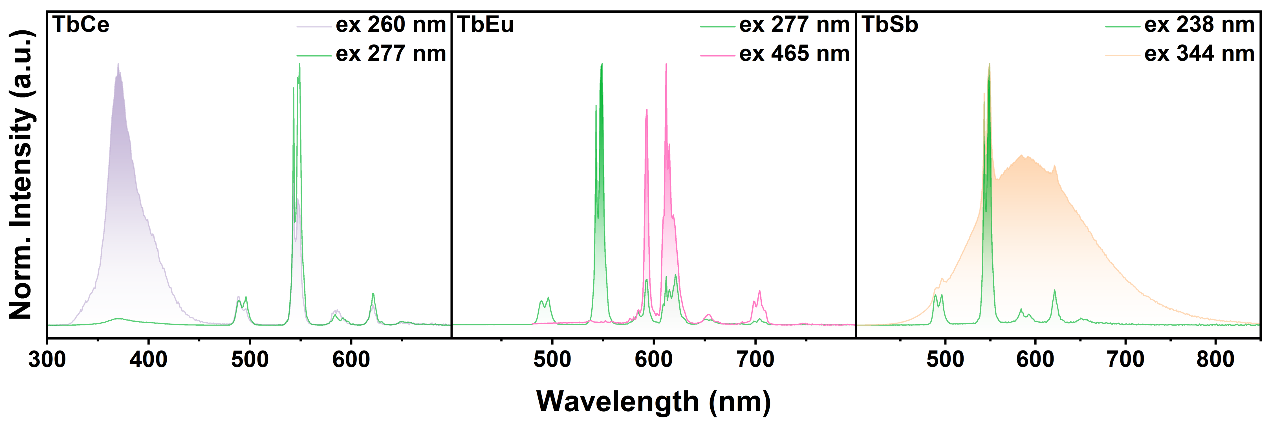


**Figure S11.** PL spectra of the S-HPDTbCl crystals doped with Ce^3+^, Eu^3+^, and Sb^3+^.


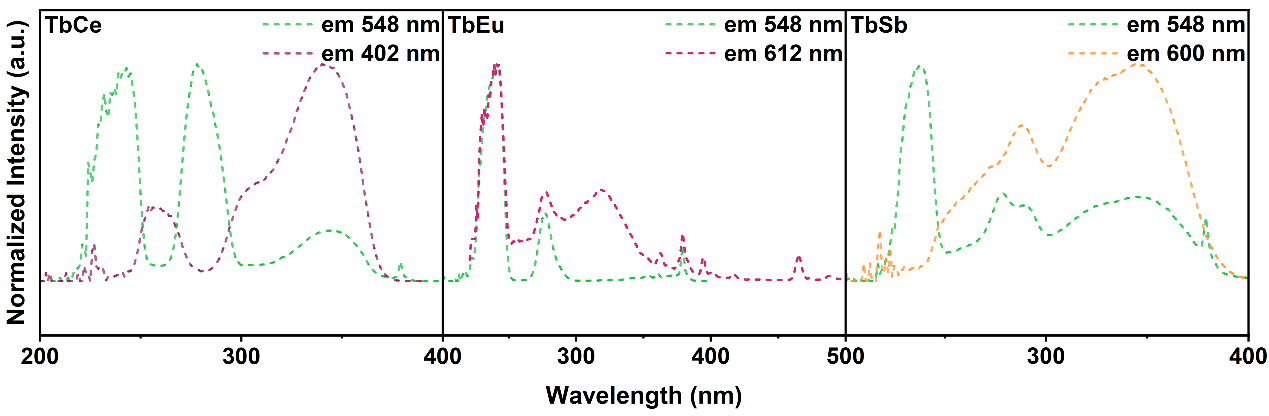


**Figure S12.** PLE spectra of the S-HPDTbCl crystals doped with Ce^3+^, Eu^3+^, and Sb^3+^.

**Table S1.** Crystal data and structure refinement of (R-HPD)_4_TbCl_7_∙MeOH.

| Empirical formula | C_21_H_52_Cl_7_N_4_O_5_Tb |
| --- | --- |
| Formula weight | 847.73 |
| Temperature/K | 150.00 |
| Crystal system | triclinic |
| Space group | P1 |
| a/Å | 7.6957(3) |
| b/Å | 9.5841(3) |
| c/Å | 12.1093(4) |
| α/° | 83.4030(10) |
| β/° | 74.0900(10) |
| γ/° | 89.9100(10) |
| Volume/Å^3^ | 852.82(5) |
| Z | 1 |
| ρ_calc_g/cm^3^ | 1.651 |
| μ/mm^‑1^ | 2.657 |
| F(000) | 430.0 |
| Crystal size/mm^3^ | 0.2 × 0.15 × 0.1 |
| Radiation | MoKα (λ = 0.71073) |
| 2Θ range for data collection/° | 5.21 to 52.962 |
| Index ranges | -9 ≤ h ≤ 9, -12 ≤ k ≤ 12, -15 ≤ l ≤ 15 |
| Reflections collected | 22382 |
| Independent reflections | 6732 [R_int_ = 0.0196, R_sigma_ = 0.0272] |
| Data/restraints/parameters | 6732/4/349 |
| Goodness-of-fit on F^2^ | 1.068 |
| Final R indexes [I>=2σ (I)] | R_1_ = 0.0141, wR_2_ = 0.0349 |
| Final R indexes [all data] | R_1_ = 0.0142, wR_2_ = 0.0349 |
| Largest diff. peak/hole / e Å^-3^ | 0.23/-0.41 |
| Flack parameter | -0.004(3) |

**Table S2.** Crystal data and structure refinement of (S-HPD)_4_TbCl_7_∙MeOH.

| Empirical formula | C_21_H_52_Cl_7_N_4_O_5_Tb |
| --- | --- |
| Formula weight | 847.73 |
| Temperature/K | 150.00 |
| Crystal system | triclinic |
| Space group | P1 |
| a/Å | 7.6954(2) |
| b/Å | 9.5868(3) |
| c/Å | 12.1117(4) |
| α/° | 83.3940(10) |
| β/° | 74.1060(10) |
| γ/° | 89.8820(10) |
| Volume/Å^3^ | 853.26(4) |
| Z | 1 |
| ρ_calc_g/cm^3^ | 1.650 |
| μ/mm^‑1^ | 2.656 |
| F(000) | 430.0 |
| Crystal size/mm^3^ | 0.2 × 0.15 × 0.1 |
| Radiation | MoKα (λ = 0.71073) |
| 2Θ range for data collection/° | 4.28 to 53.152 |
| Index ranges | -9 ≤ h ≤ 9, -12 ≤ k ≤ 12, -15 ≤ l ≤ 15 |
| Reflections collected | 24901 |
| Independent reflections | 6974 [R_int_ = 0.0233, R_sigma_ = 0.0306] |
| Data/restraints/parameters | 6974/4/349 |
| Goodness-of-fit on F^2^ | 1.000 |
| Final R indexes [I>=2σ (I)] | R_1_ = 0.0168, wR_2_ = 0.0354 |
| Final R indexes [all data] | R_1_ = 0.0173, wR_2_ = 0.0355 |
| Largest diff. peak/hole / e Å^-3^ | 0.43/-0.30 |
| Flack parameter | 0.002(3) |

**Table S3.** PLQY of previously reported ML materials

| Materials | PLQY/% | Ref. |
| --- | --- | --- |
| FPy | 54 | [7] |
| Sr_2_MgSi_2_O_7_:Dy^3+^ | 57 | [8] |
| BTB-OC4-Y | 76 | [9] |
| LiTaO_3_:0.6%Tb^3+^ | 89 | [10] |
| Zn-TPPA | 93 | [11] |
| R/S-HPDTbCl | ~100 | This Work |

**Table S4.** Crystal data and structure refinement of (4-HPD)_4_TbCl_7_.

| Empirical formula | C_20_H_48_Cl_7_N_4_O_4_Tb |
| --- | --- |
| Formula weight | 815.69 |
| Temperature/K | 150.00 |
| Crystal system | monoclinic |
| Space group | P2_1_/n |
| a/Å | 11.9895(4) |
| b/Å | 9.6127(3) |
| c/Å | 28.4427(9) |
| α/° | 90 |
| β/° | 90.7010(10) |
| γ/° | 90 |
| Volume/Å^3^ | 3277.82(18) |
| Z | 4 |
| ρ_calc_g/cm^3^ | 1.653 |
| μ/mm^‑1^ | 2.760 |
| F(000) | 1648.0 |
| Crystal size/mm^3^ | 0.2 × 0.15 × 0.1 |
| Radiation | MoKα (λ = 0.71073) |
| 2Θ range for data collection/° | 4.472 to 52.998 |
| Index ranges | -15 ≤ h ≤ 14, -12 ≤ k ≤ 12, -35 ≤ l ≤ 35 |
| Reflections collected | 69061 |
| Independent reflections | 6748 [R_int_ = 0.0342, R_sigma_ = 0.0166] |
| Data/restraints/parameters | 6748/1/327 |
| Goodness-of-fit on F^2^ | 1.065 |
| Final R indexes [I>=2σ (I)] | R_1_ = 0.0213, wR_2_ = 0.0522 |
| Final R indexes [all data] | R_1_ = 0.0277, wR_2_ = 0.0549 |
| Largest diff. peak/hole / e Å^-3^ | 0.40/-0.47 |

**Table S5.** Crystal data and structure refinement of (S-HPD)_4_EuCl_7_∙MeOH.

| Empirical formula | C_21_H_52_Cl_7_EuN_4_O_5_ |
| --- | --- |
| Formula weight | 840.77 |
| Temperature/K | 150.00 |
| Crystal system | triclinic |
| Space group | P1 |
| a/Å | 7.6934(4) |
| b/Å | 9.5891(5) |
| c/Å | 12.1081(7) |
| α/° | 83.312(2) |
| β/° | 74.204(3) |
| γ/° | 89.956(2) |
| Volume/Å^3^ | 853.22(8) |
| Z | 1 |
| ρ_calc_g/cm^3^ | 1.636 |
| μ/mm^‑1^ | 13.144 |
| F(000) | 428.0 |
| Crystal size/mm^3^ | 0.2 × 0.15 × 0.1 |
| Radiation | GaKα (λ = 1.34139) |
| 2Θ range for data collection/° | 6.648 to 114.274 |
| Index ranges | -9 ≤ h ≤ 9, -11 ≤ k ≤ 11, -15 ≤ l ≤ 15 |
| Reflections collected | 8885 |
| Independent reflections | 6034 [R_int_ = 0.0622, R_sigma_ = 0.1253] |
| Data/restraints/parameters | 6034/29/347 |
| Goodness-of-fit on F^2^ | 0.810 |
| Final R indexes [I>=2σ (I)] | R_1_ = 0.0481, wR_2_ = 0.1050 |
| Final R indexes [all data] | R_1_ = 0.0516, wR_2_ = 0.1070 |
| Largest diff. peak/hole / e Å^-3^ | 1.96/-1.46 |
| Flack parameter | 0.012(7) |

**Table S6.** The calculated total number of electrons, valence electrons, and net electrons for each atom in the (S-HPD)_4_EuCl_7_∙MeOH unit structure.

|  | Total electron number | Valence electron number | Net electron number |
| --- | --- | --- | --- |
| H1 | 1.076835 | 1 | 0.076835 |
| H2 | 1.15164 | 1 | 0.15164 |
| H3 | 0.440334 | 1 | -0.559666 |
| H4 | 0.407239 | 1 | -0.592761 |
| H5 | 0 | 1 | -1 |
| H6 | 0 | 1 | -1 |
| H7 | 0.941402 | 1 | -0.058598 |
| H8 | 1.0009 | 1 | 0.0009 |
| H9 | 0.91452 | 1 | -0.08548 |
| H10 | 1.053907 | 1 | 0.053907 |
| H11 | 0.868668 | 1 | -0.131332 |
| H12 | 0.908114 | 1 | -0.091886 |
| H13 | 0.930776 | 1 | -0.069224 |
| H14 | 0.968847 | 1 | -0.031153 |
| H15 | 0 | 1 | -1 |
| H16 | 0.225313 | 1 | -0.774687 |
| H17 | 1.00598 | 1 | 0.00598 |
| H18 | 1.042274 | 1 | 0.042274 |
| H19 | 0.931419 | 1 | -0.068581 |
| H20 | 1.058981 | 1 | 0.058981 |
| H21 | 0.988405 | 1 | -0.011595 |
| H22 | 0.972996 | 1 | -0.027004 |
| H23 | 0.940333 | 1 | -0.059667 |
| H24 | 0.437303 | 1 | -0.562697 |
| H25 | 0.335453 | 1 | -0.664547 |
| H26 | 1.038204 | 1 | 0.038204 |
| H27 | 0.496017 | 1 | -0.503983 |
| H28 | 0.434532 | 1 | -0.565468 |
| H29 | 0 | 1 | -1 |
| H30 | 0.925941 | 1 | -0.074059 |
| H31 | 0.929685 | 1 | -0.070315 |
| H32 | 0.9305 | 1 | -0.0695 |
| H33 | 1.057559 | 1 | 0.057559 |
| H34 | 0.945645 | 1 | -0.054355 |
| H35 | 0.978785 | 1 | -0.021215 |
| H36 | 1.025577 | 1 | 0.025577 |
| H37 | 0.975912 | 1 | -0.024088 |
| H38 | 0.922826 | 1 | -0.077174 |
| H39 | 0.941655 | 1 | -0.058345 |
| H40 | 1.044632 | 1 | 0.044632 |
| H41 | 1.096307 | 1 | 0.096307 |
| H42 | 1.115005 | 1 | 0.115005 |
| H43 | 1.015159 | 1 | 0.015159 |
| H44 | 0.971229 | 1 | -0.028771 |
| H45 | 0.998799 | 1 | -0.001201 |
| H46 | 1.042588 | 1 | 0.042588 |
| H47 | 0.992673 | 1 | -0.007327 |
| H48 | 0.948774 | 1 | -0.051226 |
| H49 | 0.987326 | 1 | -0.012674 |
| H50 | 0.973689 | 1 | -0.026311 |
| H51 | 0.38903 | 1 | -0.61097 |
| H52 | 0.432549 | 1 | -0.567451 |
| C1 | 3.775026 | 4 | -0.224974 |
| C2 | 3.666969 | 4 | -0.333031 |
| C3 | 3.95278 | 4 | -0.04722 |
| C4 | 3.768459 | 4 | -0.231541 |
| C5 | 3.731286 | 4 | -0.268714 |
| C6 | 3.471108 | 4 | -0.528892 |
| C7 | 3.565963 | 4 | -0.434037 |
| C8 | 3.737506 | 4 | -0.262494 |
| C9 | 3.45759 | 4 | -0.54241 |
| C10 | 3.716551 | 4 | -0.283449 |
| C11 | 3.948846 | 4 | -0.051154 |
| C12 | 3.528487 | 4 | -0.471513 |
| C13 | 3.900776 | 4 | -0.099224 |
| C14 | 3.704988 | 4 | -0.295012 |
| C15 | 3.580537 | 4 | -0.419463 |
| C16 | 3.808896 | 4 | -0.191104 |
| C17 | 3.794313 | 4 | -0.205687 |
| C18 | 3.641463 | 4 | -0.358537 |
| C19 | 3.903894 | 4 | -0.096106 |
| C20 | 3.523254 | 4 | -0.476746 |
| C21 | 3.958276 | 4 | -0.041724 |
| N1 | 6.209921 | 4 | 2.209921 |
| N2 | 6.283966 | 4 | 2.283966 |
| N3 | 6.134759 | 4 | 2.134759 |
| N4 | 6.236371 | 4 | 2.236371 |
| O1 | 7.548641 | 6 | 1.548641 |
| O2 | 7.540657 | 6 | 1.540657 |
| O3 | 7.514713 | 6 | 1.514713 |
| O4 | 7.334242 | 6 | 1.334242 |
| O5 | 7.531491 | 6 | 1.531491 |
| Cl1 | 7.728042 | 7 | 0.728042 |
| Cl2 | 7.73644 | 7 | 0.73644 |
| Cl3 | 7.726708 | 7 | 0.726708 |
| Cl4 | 7.726675 | 7 | 0.726675 |
| Cl5 | 7.735241 | 7 | 0.735241 |
| Cl6 | 7.73017 | 7 | 0.73017 |
| Cl7 | 7.801844 | 7 | 0.801844 |
| Eu | 15.150905 | 17 | -1.849095 |

**Table S7.** The calculated total number of electrons, valence electrons, and net electrons for each atom in the (S-HPD)_4_TbCl_7_∙MeOH unit structure.

|  | Total electron number | Valence electron number | Net electron number |
| --- | --- | --- | --- |
| H1 | 0.982946 | 1 | -0.017054 |
| H2 | 0.895267 | 1 | -0.104733 |
| H3 | 1.02339 | 1 | 0.02339 |
| H4 | 1.021663 | 1 | 0.021663 |
| H5 | 1.072843 | 1 | 0.072843 |
| H6 | 1.132229 | 1 | 0.132229 |
| H7 | 1.022288 | 1 | 0.022288 |
| H8 | 1.001063 | 1 | 0.001063 |
| H9 | 0.958811 | 1 | -0.041189 |
| H10 | 1.028267 | 1 | 0.028267 |
| H11 | 1.008189 | 1 | 0.008189 |
| H12 | 0.149797 | 1 | -0.850203 |
| H13 | 0 | 1 | -1 |
| H14 | 0.937911 | 1 | -0.062089 |
| H15 | 0.140017 | 1 | -0.859983 |
| H16 | 1.04529 | 1 | 0.04529 |
| H17 | 0.964844 | 1 | -0.035156 |
| H18 | 0.970642 | 1 | -0.029358 |
| H19 | 0.975266 | 1 | -0.024734 |
| H20 | 0.897244 | 1 | -0.102756 |
| H21 | 0.958587 | 1 | -0.041413 |
| H22 | 0.117677 | 1 | -0.882323 |
| H23 | 0.932247 | 1 | -0.067753 |
| H24 | 1.09866 | 1 | 0.09866 |
| H25 | 0.920521 | 1 | -0.079479 |
| H26 | 0.95379 | 1 | -0.04621 |
| H27 | 0.865483 | 1 | -0.134517 |
| H28 | 0.979775 | 1 | -0.020225 |
| H29 | 0.433485 | 1 | -0.566515 |
| H30 | 0.373488 | 1 | -0.626512 |
| H31 | 0.480278 | 1 | -0.519722 |
| H32 | 0.467357 | 1 | -0.532643 |
| H33 | 0 | 1 | -1 |
| H34 | 0.472465 | 1 | -0.527535 |
| H35 | 0.452963 | 1 | -0.547037 |
| H36 | 0.936978 | 1 | -0.063022 |
| H37 | 1.02741 | 1 | 0.02741 |
| H38 | 1.135988 | 1 | 0.135988 |
| H39 | 0.906482 | 1 | -0.093518 |
| H40 | 1.052626 | 1 | 0.052626 |
| H41 | 1.009711 | 1 | 0.009711 |
| H42 | 1.076666 | 1 | 0.076666 |
| H43 | 1.016098 | 1 | 0.016098 |
| H44 | 0.852359 | 1 | -0.147641 |
| H45 | 1.065388 | 1 | 0.065388 |
| H46 | 1.012788 | 1 | 0.012788 |
| H47 | 1.087621 | 1 | 0.087621 |
| H48 | 1.022599 | 1 | 0.022599 |
| H49 | 0.421508 | 1 | -0.578492 |
| H50 | 0.378028 | 1 | -0.621972 |
| H51 | 0.923227 | 1 | -0.076773 |
| H52 | 0.990427 | 1 | -0.009573 |
| C1 | 3.684026 | 4 | -0.315974 |
| C2 | 3.323903 | 4 | -0.676097 |
| C3 | 3.795244 | 4 | -0.204756 |
| C4 | 3.980184 | 4 | -0.019816 |
| C5 | 3.899502 | 4 | -0.100498 |
| C6 | 3.579238 | 4 | -0.420762 |
| C7 | 3.93571 | 4 | -0.06429 |
| C8 | 3.62677 | 4 | -0.37323 |
| C9 | 3.731807 | 4 | -0.268193 |
| C10 | 3.550952 | 4 | -0.449048 |
| C11 | 3.591197 | 4 | -0.408803 |
| C12 | 3.796254 | 4 | -0.203746 |
| C13 | 3.53143 | 4 | -0.46857 |
| C14 | 3.539457 | 4 | -0.460543 |
| C15 | 3.767582 | 4 | -0.232418 |
| C16 | 3.650557 | 4 | -0.349443 |
| C17 | 3.836216 | 4 | -0.163784 |
| C18 | 3.717278 | 4 | -0.282722 |
| C19 | 3.872255 | 4 | -0.127745 |
| C20 | 3.833434 | 4 | -0.166566 |
| C21 | 3.715696 | 4 | -0.284304 |
| N1 | 6.24041 | 4 | 2.24041 |
| N2 | 6.131655 | 4 | 2.131655 |
| N3 | 6.146419 | 4 | 2.146419 |
| N4 | 6.258919 | 4 | 2.258919 |
| O1 | 7.385514 | 6 | 1.385514 |
| O2 | 7.574853 | 6 | 1.574853 |
| O3 | 7.387514 | 6 | 1.387514 |
| O4 | 7.418659 | 6 | 1.418659 |
| O5 | 7.533349 | 6 | 1.533349 |
| Cl1 | 7.73564 | 7 | 0.73564 |
| Cl2 | 7.734679 | 7 | 0.734679 |
| Cl3 | 7.737142 | 7 | 0.737142 |
| Cl4 | 7.736819 | 7 | 0.736819 |
| Cl5 | 7.734914 | 7 | 0.734914 |
| Cl6 | 7.745502 | 7 | 0.745502 |
| Cl7 | 7.794516 | 7 | 0.794516 |
| Tb | 17.094162 | 19 | -1.905838 |

**Table S8.** ICP-MS elemental quantitative analysis of (S-HPD)_4_TbCl_7_∙MeOH crystals doped with Ce^3+^, Eu^3+^, and Sb^3+^.

| Samples | Nominal concentration/mol% (dopant) | Actual concentration/mol% (dopant) |
| --- | --- | --- |
| S-HPD_4_TbCl_7_:Ce | 10 | 9.3820 |
| S-HPD_4_TbCl_7_:Eu | 10 | 8.1255 |
| S-HPD_4_TbCl_7_:Sb | 10 | 9.2293 |

**References**

[1] G. Kresse,J. Furthmüller, *Comp. Mater. Sci.* **1996,** *6* (1), 15-50.

[2] G. Kresse,D. Joubert, *Phys. Rev. B* **1999,** *59* (3), 1758.

[3] P. E. Blöchl, *Phys. Rev. B* **1994,** *50* (24), 17953.

[4] J. P. Perdew,K. Burke,M. Ernzerhof, *Phys. Rev. Lett.* **1996,** *77* (18), 3865.

[5] J. D. Pack,H. J. Monkhorst, *Phys. Rev. B* **1977,** *16* (4), 1748.

[6] S. Grimme, *J. Comput. Chem.* **2006,** *27* (15), 1787-1799.

[7] Z. F. Liu,X. Y. Ye,L. H. Chen,L. Y. Niu,W. J. Jin,S. D. Zhang,Q. Z. Yang, *Angew. Chem. Int. Ed.* **2024,** *63* (8).

[8] J. C. Luo,B. Y. Ren,X. H. Zhang,M. J. Zhu,T. L. Liang,Z. F. Huang,Y. T. Zheng,X. Li,J. W. Li,Z. T. Zheng,B. Chen,Y. Fu,D. Tu,Y. Wang,Y. M. Jia,D. F. Peng, *Adv. Sci.* **2024,** *11* (1).

[9] K. Chang,J. Q. Gu,L. K. Yuan,J. F. Guo,X. X. Wu,Y. Y. Fan,Q. Y. Liao,G. G. Ye,Q. Q. Li,Z. Li, *Adv. Mater.* **2024,** *36* (38).

[10] H. Yang,Y. Wei,H. N. Ju,X. R. Huang,J. Li,W. Wang,D. F. Peng,D. Tu,G. G. Li, *Adv. Mater.* **2024,** *36* (26).

[11] X. Ma,X. J. Xu,F. N. Duan,W. Huang,Q. Chen,D. Y. Wu, *Adv. Opt. Mater.* **2022,** *10* (3).
